# Effect of methotrexate/vitamin B_12_ on DNA methylation as a potential factor in leukemia treatment-related neurotoxicity

**DOI:** 10.2217/epi-2016-0165

**Published:** 2017-08-15

**Authors:** Victoria J Forster, Alex McDonnell, Rachel Theobald, Jill A McKay

**Affiliations:** 1 Northern Institute for Cancer Research, Newcastle University, Newcastle upon Tyne, UK; 2 Institute of Health & Society, Human Nutrition Research Centre, Newcastle University, Newcastle upon Tyne, UK

**Keywords:** childhood acute lymphoblastic leukemia, epigenetic, late effects, methotrexate, neurocognition, neurotoxicity, one carbon metabolism, vitamin B_12_

## Abstract

Methotrexate (MTX) is administered to treat childhood acute lymphoblastic leukemia (ALL). It acts by inhibiting dihydrofolate reductase which reduces methyltetrahydrofolate, a key component in one carbon metabolism, thus reducing cell proliferation. Further perturbations to one carbon metabolism, such as reduced vitamin B_12_ levels via the use of nitrous oxide for sedation during childhood ALL treatment, may increase neurotoxicity risk. With B_12_ as an enzymatic cofactor, methyltetrahydrofolate is essential to produce methionine, which is critical for DNA methylation. We investigated global and gene specific DNA methylation in neuronal cell lines in response to MTX treatment and vitamin B_12_ concentration individually, and in combination. **Results:** MTX treatment alone significantly increased LINE-1 methylation in SH-SY5Y (p = 0.040) and DAOY (p < 0.001), and increased FKBP5 methylation in MO3.13 cells (p = 0.009). **Conclusion:** We conclude that altered DNA methylation of brain/central nervous system cells could be one mechanism involved in MTX treatment-related neurotoxicities and neurocognitive late effects in ALL survivors.

One carbon metabolism, which is driven by dietary B vitamins folate, B_2_, B_6_ and B_12_, is integral to DNA synthesis and the methylation of biological molecules including DNA (Figure 1) [1]. Disruption of this metabolic pathway can therefore inhibit cell proliferation, making this pathway an ideal target for cancer treatments. Methotrexate (MTX) is an antifolate drug, frequently administered to treat many types of cancers, including childhood acute lymphoblastic leukemia (ALL) and some autoimmune disorders such as arthritis. It influences one carbon metabolism (OCM) by reducing levels of tetrahydrofolate (THF) via inhibition of dihydrofolate reductase, thus reducing the production of S-adenosylmethionine (SAM), which is critical for DNA synthesis and methylation (Figure 1).

**Figure 1.**
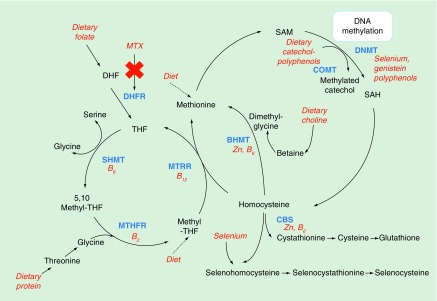
**Summary diagram of the main pathways involved in cellular one-carbon metabolism including the production of *S*-adenosylmethionine for methylation of DNA.** Dietary factors able to influence this pathway are highlighted in red, enzymes driving biological reactions are highlighted in blue/bold. The influence of methotrexate on this pathway through the inhibition of dihydrofolate reductase enzyme is highlighted in red/italics. Additional abbreviations: DHF, THF, SHMT, MTRR, MTHFR, BHMT, CBS, COMT, DNMT, SAH. BHMT: Betaine-homocysteine S-methyltransferase; CBS: Cystathione beta synthase; COMT: cystathionine beta synthase; DHF: Dihydrofolate; DHFR: Dihydrofolate reductase; DNMT: DNA methyltransferase; MTHFR: Methylenetetrahydrofolate reductase; MTRR: Methionine synthase reductase; MTX: Methotrexate; SAH: S-adenosylhomocysteine; SAM: S-adenosylmethionine; SHMT: Serine hydroxymethyltransferase; THF: Tetrahydrofolate. Modified with permission from [1] (2011).

In childhood leukemia patients, MTX treatment has been associated with acute neurotoxicities [2,3] (which in extreme cases can manifest in stroke-like symptoms) and long term [4,5] neurological issues. Furthermore, it has been noted that the use of nitrous oxide (N_2_O) in sedation during or shortly prior to administration of MTX may contribute to the risk of severe acute neurological side effects in children with acute leukemia when MTX is administered intrathecally [3]. Since, N_2_O depletes active vitamin B_12_, which is an essential cofactor in OCM, this suggests that inhibition of OCM via multiple routes in other words combined MTX treatment and N_2_O use, may have a synergistic effect [6] and increase the risk of neurotoxcity in comparison with the use of either agent alone. Indeed, in rats high dose MTX concomitant with N_2_O exposure resulted in toxicity [7]. Furthermore, a recent meta-analysis reported a significant association of the C677T polymorphism of the methylene tetrahydrofolate reductase gene which is responsible for the conversion of 5′10 methyltetrahydrofolate (MTHF) to MTHF in OCM, with overall MTX toxicity, hepatotoxicity, hematological toxicity and neurotoxicity [8]. Currently, the underlying biological mechanisms behind these associations are unknown.

DNA methylation, (addition of a methyl group to cytosine followed by guanine [CpG sites]), is one mechanism of gene regulation. It is generally accepted that methylation of CpG rich regions inhibits binding of the regulatory machinery, leading to gene silencing. Consequently gene dysregulation via aberrant DNA methylation may be an important contributor to disease. Also, while the DNA sequence is generally more resistant to change in response to noncarcinogenic environmental factors, DNA methylation is more plastic and is altered more easily by a variety of more subtle exposures [9]. Evidence suggests that DNA methylation may play an important role in neurotoxicities such as cognitive impairments (reviewed in [10]). In addition, DNA methylation has recently been suggested to play a key role in gene regulation during peripheral nerve myelination [11]. Malformation or destruction of myelin sheaths leads to motor and sensory disabilities, suggesting a potential role for DNA methylation in neurological disorders [12]. Furthermore, MTX treatment has been reported to alter DNA methylation in patients being treated for arthritis [13–15]. We therefore, hypothesize that DNA methylation may be one potential mediating mechanism linking MTX treatment with increased risk of neurotoxicity. Furthermore, we suggest that vitamin B_12_ levels may influence the effect MTX treatment has on DNA methylation patterns.

To test the initial hypothesis that MTX and B_12_ treatments may have the potential to alter DNA methylation in cell lines derived from cell populations which maybe relevant for neurotoxicity, we used a 3 × 3 factorial study design we treated neuronal and oligodendrocyte cell lines with varying concentrations of MTX in combination with different concentrations of vitamin B_12_. After 3 days exposure we harvested cells for DNA extraction to measure both global and gene specific DNA methylation in response to treatments.

## Materials & methods

### Cell lines & tissue culture

Oligodendrocytic MO3.13 (Cedarlane Laboratories, Canada), medulloblastoma DAOY and neuroblastoma SH-SY5Y cell lines (ATCC, Middlesex, UK) were all cultured in DMEM high glucose (Sigma) with 10% of dialysed fetal bovine serum (dFBS) and 1% penicillin/streptomycin solution (Sigma). Dialyzed FBS (ThermoFisher Scientific) is depleted of small molecules (less than 10 kDa) including vitamin B_12_, allowing for more defined culture conditions than with conventional FBS, where components can be reintroduced as required. Cells were routinely passaged every 2–3 days when reaching 80–90% confluence. Briefly, all media were removed from culture vessels and cells were washed once with prewarmed PBS. The 1× trypsin (Sigma), diluted in PBS was added and cells were incubated at 37°C for 5 min to detach them from the plastic. Flask was tapped gently to detach cells and growth medium was immediately added before gently pipetting cells up and down to create a single-cell suspension before quantification of cell number.

### Methotrexate & vitamin B_12_ treatment of cells for proliferation analysis

**Figure 2.**
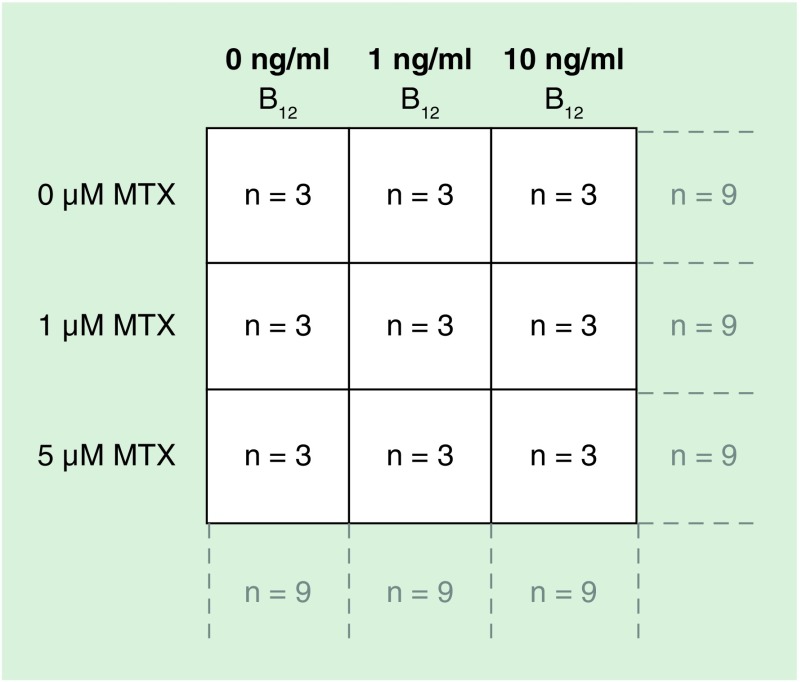
**Schema depicting 3 × 3 factorial study design.** The experiment was carried out in triplicate for each cell line, resulting in an n = 9 per group to investigate the main effects of MTX and vitamin B_12_ treatments alone, and n = 3 to investigate the interaction between treatments. MTX: Methotrexate.

To ensure selected doses of MTX reduced proliferation in all three-cell lines, initial growth proliferation experiments were performed. MO3.13, DAOY and SH-SY5Y were seeded in T25 cm^2^ flasks at the following densities (total cell number): MO3.13 and DAOY; 1 × 10^5^ - SH-SY5Y - 2 × 10^5^. In a 3 × 3 factorial design, cells were treated with various concentrations of vitamin B_12_ (Sigma) (0, 1 and 10 ng/ml final concentration in culture media) at the time of seeding and left to adhere overnight before a single dose treatment with either 0, 1 or 5 μM MTX (Sigma), resulting in nine overall treatment groups (see Figure 2). The experiment was carried out in triplicate for each cell line, resulting in an n = 9 per group to investigate the main effects of MTX and vitamin B_12_ treatments alone, and n = 3 to investigate the interaction between treatments (Figure 2). Both MTX and vitamin B_12_ were resuspended in molecular grade water. MTX was incubated with the cells for the remainder of the culture period of 3 days, at 37°C in a 5% CO_2_ incubator before quantifying cells with a hemocytometer.

### Quantification of cells using a hemocytometer

Cells were quantified using a counting chamber (Nebauer) after mixing in a 1:1 ratio with trypan blue (Sigma) to exclude dead cells. The average of two separate counts was taken to calculate cell number.

### Methotrexate & vitamin B_12_ treatment of cells for methylation analysis

Cells were seeded at higher densities proportional to the original growth proliferation experiments in order to ensure adequate DNA for methylation assays in T175 cm^2^ flasks at the following starting densities (total cell number): MO3.13 and DAOY; 7 × 10^5^ – SH-SY5Y – 14 × 10^5^ cells were treated with MTX and B_12_ at the same final concentrations and under the same conditions as detailed above. Three days post MTX dosing, cells were washed twice in flasks with PBS, detached using 1× trypsin in PBS, followed by inactivating trypsin with 1 ml of DMEM 10% media (detailed above), centrifuged at 300 × *g* for 5 min and then proceeding to DNA extraction. The experiment was carried out in triplicate for each cell line, resulting in an n = 9 per group to investigate the main effects of MTX and vitamin B_12_ treatments alone, and n = 3 to investigate the interaction between treatments (Figure 2).

### DNA extraction, bisulphite modification & DNA methylation assessment using pyrosequencing

DNA was extracted and purified from cell pellets using the using an EZNA tissue DNA isolation kit (Omega Biotek) as detailed in manufacturer’s protocol. DNA purity and concentration was determined using a Nanodrop Spectrophotometer (ThermoScientific).

Bisulfite conversion of DNA was performed using EZ DNA Methylation Gold™ kit (Zymo Research) as detailed in manufacturers’ protocol. Briefly, 1 μg of genomic DNA was incubated with CT conversion reagent and incubated at the following temperatures; 98°C for 10 min, 64°C for 2.5 h, held at 4°C. DNA was then transferred to a spin column, washed, desulphonated and purified, finally eluting in a 12 μl volume of elution buffer and stored routinely at -20°C.

We used quantitative bisulfite pyrosequencing to determine the percentage methylation at individual CpG sites within the *LINE1* [16], *HOXA4* [17] and *FKBP5* loci. *LINE1* methylation was investigated as a proxy for genome-wide methylation changes [16]. *HOXA4* methylation was investigated as increased methylation at this locus has previously been observed in response to exposure to therapy in acute lymphoblastic leukemia patients [18] and therefore methylation at this loci may be altered in response to MTX treatment. *HOXA4* methylation was measured +32 to +219 bp upstream of the transcriptional start site within a CpG island spanning the transcriptional start site, with methylation of this region previously having been associated with loss of gene expression [17]. *FKBP5* methylation was investigated as this gene is known to exhibit altered DNA methylation in abused children and to be associated with adult onset depression [19] an adverse effect associated with childhood cancer [20–22]. Methylation of *FKBP5* was measured at a glucocorticoid responsive element in intron seven of the gene. While there have been no reports of altered methylation at this region being associated with altered expression of the gene itself, demethylation in this region has been reported to increase stress dependant gene transcription leading to long-term dysregulation of glucocorticoid regulation [19]. Briefly, 1 μl of bisulfite treated DNA was added as a template in PCR reaction using either 10 or 12.5 μl Hot Star Taq mastermix (Qiagen), in a total volume either 20 or 25 μl, respectively. All primer sequences and PCR conditions are shown in Table 1. Amplification was carried out in either a ThermoHybaid PxE0.2 (ThermoScientific) or Veriti (Applied Biosystems) 96 well Thermal Cycler using the following protocol for *LINE1*; 95°C 15 min, then 50 cycles of 95°C 15 s, annealing temperature 30 s, 72°C 15 s, followed by 72°C for 5 min, or 95°C 15 min, then 40 cycles of 95°C 30 s, annealing temperature 30 s, 72°C 1 min, followed by 72°C for 7 min for *HOXA4* and *FKBP5* respectively. Biotin-labeled PCR products were captured with Streptavidin Sepharose beads (GE Healthcare), and made single stranded using a Pyrosequencing Vacuum Prep Tool (Qiagen). Sequencing primers were annealed to the single stranded PCR product by heating to 80°C, followed by slow cooling. Pyrosequencing was then carried out on a Pyromark MD system. Cytosine methylation was quantified by the Pyro Q CpG 1.0.6 software.

**Table 1.** **Primer sequences and PCR and Pyrosequencing^®^ conditions.**

**Gene**	**Forward primer**	**Reverse primer**	**PCR**					**Pyrosequencing**	**Ref.**

			**PCR Volume (μl)**	**Primer Concentration (ng)**	**Size (bp)**	**Annealing (°C)**	**Magnesium (μl)**	**Sequencing primer**	
*LINE1*	TTTTGAGTTAGGTGTGGGATATA	Biotin-AAAATCAAAAAATTCCCTTTC	20	10	168	50	0	AGTTAGGTGTGGGATATAGT	[16]

*HOXA4*	TACACTTCACAAATTAATAACCATAAACTC	Biotin -GTTGTTGTAGYGGTAGGTGTTG	25	75	188	63	1.5	AACCCAAATTCCCTCCCTT	[17]

*FKBP5*	ATTTGTAGTTGGGATAATAATTTGG	Biotin-AAATAAAATACAACTTTTCAAAACTAA	25	75	240	58	1.5	TGGAGTTATAGTGTAGGTTTT	

### Statistical analysis

Analysis of variance was used to examine the main effects of MTX treatment and vitamin B_12_ concentration, and the interaction between these treatments on cell proliferation and mean DNA methylation for individual genes (calculated as a mean methylation value across the analyzed CpG site for each gene loci; 3 CpGs for *LINE1*, 4 CpGs for *HOXA4* and 2 CpGs for *FKBP5*), using Bonferroni post hoc tests to examine the difference between MTX and vitamin B_12_ concentrations on DNA methylation. A p-value of <0.05 was considered to be statistically significant for main effects of treatment, interaction and Bonferroni post hoc tests.

## Results

### Influence of MTX treatment & vitamin B_12_ concentration on cell proliferation

**Figure 3.**
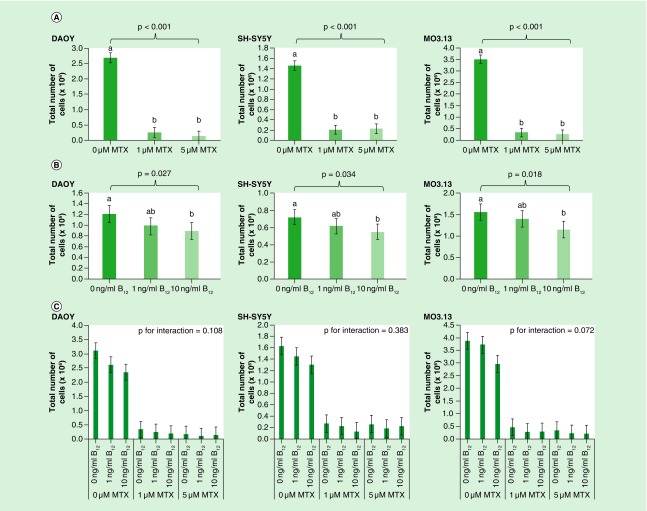
**Effect of (A) MTX treatment (B) vitamin B_12_ concentration and (C) interaction between treatments on proliferation of DAOY, SH-SY5Y and MO3.13 cells 4 days after seeding.** Cells were seeded in T25 cm^2^ flasks at the following starting densities (total cell number): MO3.13 and DAOY; 1 × 10^5^ - SH-SY5Y - 2 × 10^5^. Data were analyzed using univariate ANOVA, n = 9 per treatment group for main effects (i.e., MTX and vitamin B_12_ treatments) and n = 3 for interaction, error bars represent 95% CIs. P-values relate to effect of treatment on methylation; where groups do not share the same letter post hoc tests revealed significant differences (p > 0.05). MTX: Methotrexate.

MTX exposure was not cytotoxic at either dose used within the time period of the experiment. However, both 1 and 5 μM MTX significantly reduced proliferation of all cell lines when measured 3 days post-treatment compared with untreated cells (Figure 3A) and therefore exerted a growth inhibitory effect. Vitamin B_12_ concentration also significantly influenced proliferation in all cell lines, with 10 ng/ml B_12_ reducing proliferation compared with both 0 and 1 ng/ml treated cells (Figure 3B). No significant interactions were observed between MTX treatment and vitamin B_12_ concentrations for cell proliferation in any cell line investigated (Figure 3C).

### Influence of MTX treatment on DNA methylation

**Figure 4.**
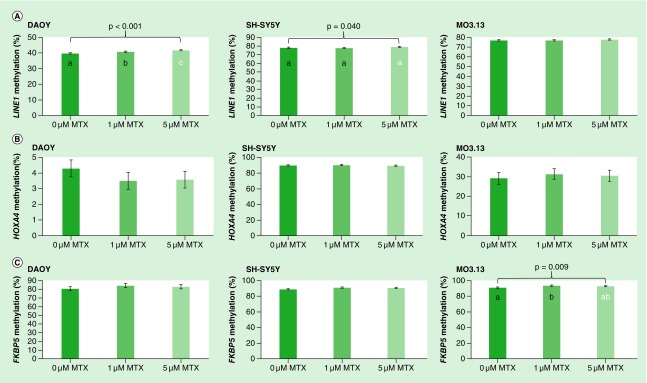
Effect of MTX treatment on (A) LINE1 (B) HOXA4 and (C) FKBP5 DNA methylation in DAOY, SH-SY5Y and MO3.13 cells. Data were analyzed using univariate ANOVA, n = 9 per treatment group*, error bars represent 95% CIs. P-values relate to effect of treatment on methylation; where groups do not share the same letter post hoc tests revealed significant differences (p > 0.05). (*with the exception of HOXA4 methylation in MO3.13 cells where n = 7, 9 and 8 respectively for 0, 1 and 5 μM MTX groups). MTX: Methotrexate.

MTX treatment increased *LINE1* methylation in DAOY, SH-SY5Y and MO3.13 cells by 2.1, 1.2 and 0.8% respectively across the dose range. These differences were found to be statistically significant for DAOY and SH-SY5Y (p < 0.001 and p = 0.040 respectively) but not MO3.13 (p = 0.102) (Figure 4A). In DAOY, methylation was increased in response to MTX treatment compared with untreated cells, with post hoc tests revealing significant increases in methylation associated with low (1 μM) and high (5 μM) levels of MTX for all CpG sites investigated (Figure 4A). In SH-SY5Y, methylation was also found to be increased in response to MTX treatment, although post hoc tests did not suggest significant differences between individual MTX treatment groups (Figure 4A).

*HOXA4* methylation was decreased (by 0.7–0.8%) in DAOY, but increased in SH-SY5Y and MO3.13 cells in response to MTX treatment (by 0.3–0.5 and 1.5–2.3% respectively), but these changes were not found to be statistically significant (Figure 4B).

*FKBP5* methylation increased in response to MTX treatment in DAOY, SH-SY5Y and MO3.13, with 3.6, 2.7 and 2.8% increases in methylation between untreated and 1 μM MTX treated cells, respectively. However, statistical significance was only reached for MO3.13 cells (Figure 4C; p = 0.009), with post hoc tests revealing significant differences between 0 and 1 μM MTX treated cells (p = 0.008 for difference in methylation between these two treatment groups), with no significant difference in methylation between 0 and 5 or 1 and 5 μM treated cells.

### Influence of vitamin B_12_ concentration on DNA methylation

Changes in vitamin B_12_ concentration had little impact on methylation changes across cell lines and loci investigated, with only one statistically significant increase in methylation observed at the *HOXA4* loci in SH-SY5Y cells (p = 0.029) (Table 2). Post hoc tests established a small significant increase in methylation by 1.13% between 0 and 10 ng/ml treated cells.

**Table 2.** **Methylation at all investigated loci in response to vitamin B_12_ treatment in DAOY, SH-SY5Y and MO3.13 cells.**

	**Vitamin B_12_ concentration (ng/ml)**			**Pooled SEM**	**p-value**

	**0**	**1**	**10**		
***DAOY***					

Mean *LINE1* methylation	40.67	40.97	40.72	0.164	0.414

Mean *HOXA4* methylation	3.92	3.79	3.63	0.255	0.731

Mean *FKBP5* methylation	81.00	84.15	83.10	1.205	0.198

***SH-SY5Y***					

Mean *LINE1* methylation	78.25	78.43	78.20	0.363	0.899

Mean *HOXA4* methylation*	88.90^a^	89.20^ab^	90.03^b^	0.588	0.029

Mean *FKBP5* methylation	91.34	90.39	89.42	1.159	0.514

***MO3.13***					

Mean *LINE1* methylation	77.22	77.09	76.73	0.272	0.436

Mean *HOXA4* methylation	30.57	29.36	31.07	1.35	0.662

Mean *FKBP5* methylation	92.55	92.31	92.73	0.569	0.873

Data were analyzed using univariate ANOVA, n = 9 or 8* per treatment group. P-values relate to effect of treatment on methylation; where groups do not share the same letter post hoc tests revealed significant differences (p > 0.05).

SEM: Standard error of the mean.

### Influence of an interaction between MTX treatment & vitamin B_12_ concentration on DNA methylation

**Figure 5.**
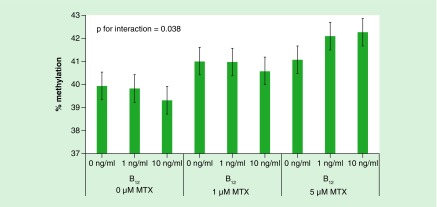
**Mean LINE1 methylation of DAOY treated with various concentrations of methotrexate and vitamin B_12_.** n = 3 per treatment group. Data were analyzed using univariate ANOVA, p = 0.038 for interaction between MTX and vitamin B_12_ treatments. Error bars represent 95% CIs. MTX: Methotrexate.

There was limited evidence to suggest an interaction between MTX and vitamin B_12_ treatments and altered DNA methylation (data not shown). Only one significant interaction between MTX and vitamin B_12_ treatments was observed whereby in DAOY cells 10 ng/ml vitamin B_12_ appeared to be protective against MTX-associated *LINE-1* methylation increases at 1 μM treatment, whereas both 1 and 10 ng/ml vitamin B_12_ concentrations increased *LINE-1* methylation in response to 5 μM MTX treatment (Figure 5). Given this finding was isolated; it may be due to chance.

## Discussion

MTX treatment has been associated with both acute [2] and long term [4,5] neurotoxicities in childhood leukemia survivors, although the underlying mechanisms are not well understood. Evidence suggests that DNA methylation may play an important role in the development of neurotoxicities associated with MTX treatment such as cognitive impairment [10,23–25] and depression [26–29]. Therefore, given that MTX is known to inhibit dihydrofolate reductase, thus reducing the production of SAM, which is critical for DNA methylation [30–32] and has been previously proposed as a plausible mechanism for MTX toxicity [32], we hypothesize that DNA methylation patterns may be altered by MTX treatment and therefore may be a potential mediating mechanism between MTX treatment and treatment-associated acute and long term neurotoxicities. As there is evidence that additional perturbations to OCM may increase the risk of MTX-associated toxicities [3,8], we further propose that vitamin B_12_ levels may influence the impact of MTX treatment on DNA methylation.

To investigate the plausibility of these hypothesizes, we tested the initial hypothesis that MTX and B_12_ treatments may have the potential to alter DNA methylation in cell lines derived from cell populations which maybe relevant for neurotoxicity. Using a 3 × 3 factorial study design we treated neuronal and oligodendrocyte cell lines concomitantly with low and high levels of MTX and vitamin B_12_ and assessed global and site specific DNA methylation in response to the treatments individually and in combination.

In support of our initial hypothesis, we observed significant increases in global DNA methylation (measured using the *LINE1* pyrosequencing assay) in DAOY and SH-SY5Y investigated in response to both low (1 μM) and high (5 μM) levels of MTX treatment. While these changes were small (i.e., in the region of 1% increase), they were statistically significant, with the pattern of methylation change was consistent across the cell lines. Similar small, significant changes are commonly reported in studies investigating the influence of environment on DNA methylation, and perhaps of more specific relevance, have been reported in response to MTX treatment in blood samples of arthritic patients [14]. Furthermore, low level MTX treatment was associated with increased mean methylation at the *FKBP5* locus in MO3.13. While an increase in methylation in response to MTX treatment, which reduces available folate, seems paradoxical, this is in line with previous reported models of methyl donor depletion [33–36] and MTX treatment [13–15,37,38]. As the folate, choline, methionine and vitamins B_6_ and B_12_ pathways are closely connected, perturbations in one of these pathways may plausibly lead to compensatory changes in others, which may result in the observed paradoxical effect on DNA methylation. Indeed in support of this hypothesis, folate-induced changes in methylation and gene expression have been previously observed to potentially influence one-carbon metabolism and methylation pathways [35,36,39]. These data add to the currently limited evidence proposing MTX treatment influences DNA methylation patterns, and suggest that MTX treatment is able to alter methylation patterns in cell types derived from those likely to be key in the development of neurotoxicity.

Previously, both global DNA hyper- and hypomethylation have been associated with MTX treatment [14,15,37,38]. While global DNA hypomethylation has been observed in osteosarcoma cells treated with 1 and 40 μM MTX for 3 days in culture [37] and in embryonic neural tissue in a mouse model of MTX-induced neural tube defects [38], global DNA hypermethylation has been observed in blood samples of arthritic patients treated with MTX compared with untreated patients [14,15]. Here, we report increased methylation in response to low and high levels of MTX treatment using *LINE1* methylation as a measure of global DNA methylation. Although, there is limited evidence for the influence of MTX treatment on gene specific methylation, Cribbs *et al.* reported reduced methylation of the *FOXP3* gene in cultured Treg cells [13], while Wang *et al.* reported 132 differentially methylated regions (35 low methylated and 97 high methylated) in embryonic neural tissue of their MTX induced neural tube defects mouse model [38]. We provide additional support for an effect of MTX treatment on DNA methylation at specific gene loci, which may have implications for gene expression and therefore MTX-related toxicity. While in the current study the implications of the altered methylation we report in response to MTX treatment in neuronal cells cannot be determined in the context of acute and long-term neurotoxicities, it is plausible that the occurrence of these, and other yet unobserved DNA methylation changes, could be a mediating mechanism leading to these, and other, adverse events associated with this treatment.

It is important to note the use of cell lines was a limitation of this study. Malignant cells often express folylpolyglutamate synthase, causing MTX to be metabolized to and retained within the cell as polyglutamates, which may have affected the cell response to the MTX. As this does not occur in normal neural tissue this may therefore limit the comparability of this model with normal cells. Furthermore, here cells were treated once with MTX over a 3 day period, whereas clinical treatments regimens for childhood leukemia utilize multiple doses of this drug given over time. Our 1 μM dose reflected a single intrathecal exposure at a clinically relevant concentration in cerebrospinal fluid (reported to be 1.1 ± 0.4 μM) [40]. However, it is highly plausible that DNA methylation marks that are susceptible to change via MTX exposure are likely to be cumulatively altered over the course of treatment protocols, and therefore may exhibit fluctuations in methylation change dependent on treatment dosing and other environmental influences. Further knowledge of such cumulative changes during the course of treatment, including whether they are sustained and long lasting, will be critical in understanding if and how DNA methylation plays a role in the development of both acute and long term neurotoxicity associated with the treatment of childhood leukemia. Therefore, exploring intermittent exposures to lower concentrations of MTX will be important in future studies.

We proposed that vitamin B_12_ levels may influence the impact of MTX treatment on DNA methylation; however our data provide limited evidence in support of this hypothesis. While *HOXA4* methylation increased in response to 10 ng/ml vitamin B_12_ compared with 0 ng/ml vitamin B_12_ in SH-SY5Y cells, and a significant interaction was observed between MTX and vitamin B_12_ treatment in DAOY cells, we observed no further evidence that vitamin B_12_ concentration alone or in combination with MTX altered DNA methylation, which may suggest that these observations were chance findings.

However, it is pertinent to highlight that this preliminary study was restricted in its assessment of DNA methylation markers which is likely to have limited the likelihood of observing methylation changes in response to treatment. Indeed, previous studies have observed global and site-specific methylation changes in response to vitamin B_12_ concentrations [41,42] and therefore further investigations are warranted to examine the potential interactions between one carbon moieties, MTX treatment and subsequent effects of DNA methylation for acute and long-term neurotoxicities.

The use of dialyzed FBS was vital for our investigation, as small molecules under 10 kDa such as vitamin B_12_ are removed from the media. However, inevitably other small molecules involved in OCM are also removed during this process (i.e., vitamin B_2_ [riboflavin], vitamin B_6_, folate and choline). We therefore used basal DMEM high glucose media to culture cells, as it lacks vitamin B_12_, but importantly contains these other vital OCM components; folic acid, riboflavin, vitamin B_6_ (in the form of pyridoxine hydrochloride) and choline (in the form of choline chloride). Hence, considering OCM is a complex process involving many small molecules, we cannot guarantee that use of dialyzed FBS and our chosen media formulation has provided physiologically relevant levels of all of these components, but suggests that our experimental design has ensured that the major components are all present, excepting vitamin B_12_. Indeed, we recognize that in particular concentrations of folic acid in the culture medium are supraphysiological (approx. 9 μM) as opposed to the normal range of plasma folate (2–21 nmol/L 5-MTHF). While the difference in form and levels of folate may influence the interpretation of these data in a clinical context, particularly given that folate deficiency is common in childhood leukemia patients, it is important to note that folate concentrations were uniform across experiments performed. Therefore, while it is plausible that both MTX and B_12_ treatments may influence DNA methylation differently in the context of physiological folate concentrations, we would hypothesize that it is likely that overall baseline methylation levels will be altered or responses to these treatments be exacerbated, rather than changed *per se*. It is important to note that for the duration of the experiment, all cell lines used experienced no negative effects on proliferation by culturing in dFBS compared with nondialyzed FBS typically recommended for the culture of these cell lines (data not shown).

In addition to investigating altered DNA methylation in response to MTX and vitamin B_12_, we monitored cell proliferation in response to these treatments. Given the cytotoxic nature of MTX, as expected both physiological relevant and high levels of treatment led to significant limitation of growth proliferation in both neuronal and oligodendrocyte cell lines. Perhaps unexpected was the observation that high doses (i.e., 10 ng/ml) of vitamin B_12_ reduced proliferation in all cell lines. We would suggest that this response may be due to exposure to supra-physiological levels of vitamin B_12_ which would not usually be encountered by these cell types *in vivo* due to regulation of circulating vitamin B_12_ levels by the liver.

## Conclusion

Here, we provide evidence in support of our hypothesis that altered methylation may potentially act as a mediating mechanism between MTX treatment and subsequent neurotoxicities, however currently the evidence is limited to support our suggestion that altered B_12_ concentrations may further influence the impact of MTX treatment on this potential mechanism. Our results are limited in investigating three cell lines, but suggest that alteration of methylation in different neural cell types at concentrations of MTX known to be reached in the cerebrospinal fluid of patients undergoing leukemia treatment [43] might be affected by MTX treatment. While the use of cell lines as opposed to human tissues is a limitation of this study, this approach was warranted in the first instance to provide proof of principle where availability of human samples are lacking (i.e., in order to determine the effect of treatment in human tissues samples collected before and after treatment would be required, in this case unethical and invasive biopsies of neural tissue).

Here, we measured DNA methylation using one global proxy measure and two gene loci. In order to fully elucidate the relationship between MTX, DNA methylation and neurotoxicity risk, further detailed knowledge of the effects of MTX and the interaction between drug treatment and various aspects of OCM (including levels of vitamin B_12_, folate and choline) on DNA methylation patterning is warranted. The utilization of genome-wide methylation assessments *in vitro* (preferably iPSC/ESC derived nonmalignant neural or primary cells as models of normal cells as opposed to tumor-derived cell lines) and animal model studies will be critical in understanding these mechanistic processes and interactions where relevant human tissue samples are not available or practicable. Such investigations will inform future molecular epidemiological investigations involving patients undergoing treatment and long-term cancer survivors to examine the potential role of key DNA methylation marks and aspects of OCM in MTX-associated acute and late neurotoxcities. The outcomes of these studies may have clinical relevance, including the potential for monitoring of one carbon moieties during MTX treatment of childhood leukemia patients to identify individuals at high risk of developing acute toxicity, or the development of robust DNA methylation biomarkers to predict acute and/or long-term toxicities. As improved treatments for childhood leukemia are leading to increasing survival rates [44], such future research is highly justified in order to aid refining of therapies and to identify high risk individuals for intervention treatments to reduce long-term adverse effects of treatment. These investigations will also provide valuable data resource to inform studies in other patients groups treated with MTX such as numerous adult cancer types and arthritis sufferers.

**Summary points**

**Background**

Methotrexate (MTX) is an antifolate drug used to treat pediatric acute lymphoblastic leukemia and works by reducing levels of tetrahydrofolate via inhibition of dihydrofolatereductase, hence interrupting one carbon metabolism and interfering with DNA synthesis and methylation.
MTX treatment in acute lymphoblastic leukemia has been associated with acute and chronic neurotoxicity and it has been proposed that levels of other factors involved in one carbon metabolism such as vitamin B_12_ may influence the risk of neurotoxicity.
The use of nitrous oxide in sedation during or shortly prior to administration of MTX may contribute to the risk of severe acute neurological side effects in children via the depletion of vitamin B_12_.
Altered methylation via depletion of S-adenosyl methionine has been suggested to have an impact on myelination, providing one possible mechanism for neurotoxicity after methotrexate treatment.

**Influence of MTX treatment & vitamin B_12_ concentration on cell proliferation**

MTX exposure was not cytotoxic at 1 and 5 μM doses within the experimental time period of 3 days, however both doses significantly reduced proliferation of all cell lines.
High doses (10 ng/ml) of vitamin B_12_ reduced proliferation of all cell lines.

**Influence of MTX treatment &/or B_12_ treatment on DNA methylation**

MTX treatment alone significantly increased *LINE-1* methylation in SH-SY5Y (p = 0.040) and DAOY (p < 0.001), but not MO3.13 cells, and increased *FKBP5* methylation in MO3.13 cells (p = 0.009).
While these changes were small (i.e., in the region of 1% increase), they were statistically significant, with the pattern of methylation change being consistent across the cell lines.
Similar small, significant changes are commonly reported in studies investigating the influence of environment on DNA methylation, and perhaps of more specific relevance, have been reported in response to MTX treatment in blood samples of arthritic patients.
There was limited evidence for an effect of vitamin B_12_ concentration alone or in combination with MTX treatment on DNA methylation within the confines of this study.

**Conclusion**

Neurotoxicity is a worrying acute side effect of childhood leukemia treatment.
Long-term neurocognitive deficits are increasingly observed in survivors, with MTX implicated as the main causative agent.
Altered methylation may act as a mediating mechanism between MTX treatment and subsequent neurotoxicities.
Currently the evidence is limited to support our suggestion that altered B_12_ concentrations may further influence the impact of MTX treatment on this potential mechanism.
Our results suggest methylation is altered in different neural cell types at concentrations of MTX known to be reached in the cerebrospinal fluid of patients undergoing leukemia treatment.
Future work on this topic should include the utilization of genome-wide methylation assessments *in vitro* such as induced-pluripotent or embryonic stem cells and animal model studies.
